# Low expression of microRNA-204 (miR-204) is associated with poor clinical outcome of acute myeloid leukemia (AML) patients

**DOI:** 10.1186/s13046-015-0184-z

**Published:** 2015-07-01

**Authors:** Aleksandra Butrym, Justyna Rybka, Dagmara Baczyńska, Andrzej Tukiendorf, Kazimierz Kuliczkowski, Grzegorz Mazur

**Affiliations:** Department of Hematology, Blood Neoplasms and Bone Marrow Transplantation, Wroclaw Medical University, Pasteur 4 Str, 50-367 Wroclaw, Poland; Department of Physiology, Wroclaw Medical University, Wroclaw, Poland; Department of Forensic Medicine, Molecular Techniques Unit, Wroclaw Medical University, Wroclaw, Poland; Department of Epidemiology, Cancer Center-Institute of Oncology, Gliwice, Poland; Department of Internal and Occupational Diseases and Hypertension, Wroclaw Medical University, Wroclaw, Poland

**Keywords:** miR-204, Acute myeloid leukemia, Expression, Survival, Prognosis

## Abstract

**Background:**

Acute myeloid leukemia (AML) is a heterogeneous neoplasm of the bone marrow with poor prognosis. In clinical practice new prognostic factors are still needed. MicroRNAs (miRs), small endogenous noncoding RNAs, play an essential role in the development and progression of acute leukemia. The aim of the study was to evaluate miR-204 expression in patients with AML at diagnosis and after induction chemotherapy, in comparison to healthy controls. We also investigated, if miR-204 expression correlates with clinical features of AML patients.

**Methods:**

miR-204 expression has been analyzed using RT-PCR in 95 bone marrow specimens from newly diagnosed AML patients in comparison to 20 healthy subject.

**Results:**

We showed down-regulated miR-204 expression in AML patients, which was associated with shorter patients’ survival. Higher expression of miR-204 in patients after induction therapy was correlated with complete remission achieving.

**Conclusions:**

We showed low miR-204 expression in AML and found it to be an independent prognostic factor in this patient population.

## Introduction

Acute myeloid leukemia (AML) in adults is a hematological malignancy with proliferation of myeloblasts in the bone marrow. Population of AML patients has heterogenous clinical course and different prognosis. Although dynamic progress on the field of pathogenesis and development of this blood cancer has been made, it is still difficult to predict clinical outcome and response to therapy of AML patients. Genetic and molecular markers are used in every day practice, but new predictors are needed, for better patients’ classification and therapy planning [1, 2].

microRNA (miRs) are small non-coding RNAs, which play an important role in neoplastic transformation. miRs act by influencing posttranscriptional gene expression, cell development, differentiation, proliferation and apoptosis [3–8].

microRNA-204 (miR-204) role has been investigated and described in few solid tumors, particularly pancreatic and colorectal cancer, where it was found to be associated with process of autophagy [3, 9]. But there is no data about miR-204 role in acute myeloid leukemia. The purpose of this study was to evaluate miR-204 expression in AML patients in relation to clinical factors, survival and comparison to healthy subjects.

## Material and methods

### Patients characteristic

The study included 95 patients (aged 60.2 ± 15.0, 22–90, Male = 61 %) with newly diagnosed AML. Samples of the bone marrow for miR-204 expression analysis were collected before start of chemotherapy and repeated after completed induction chemotherapy (in 40 patients). Patients were treated in the Department of Hematology, Blood Neoplasms and Bone Marrow Transplantation of Wroclaw Medical University, Wroclaw, Poland. A control group of 20 healthy subjects was also taken into account (aged 64.2 ± 10.5, 39–80, Male = 65 %). According to AML FAB classification, 7 patients had AML M0, 34 had M1, 29 had M2, 14 had M4 and 11 had M5. There were 73 patients with primary leukemia and 22 patients with leukemia secondary to myelodysplastic or myeloproliferative syndrome. Summary of patients’ characteristics is presented in Table 1.

**Table 1:** Clinical characteristics of patients with AML

Characteristic	Cases
Sex	
Male	56
Female	39
Age (years)	
Range	22-90
Median	61
FAB subtype	
M0	7
M1/M2	63
M4/M5	25
WBC (G/L)	
Range	0.2-295
Median	14
HGB g%	
Range	5.8-13.1
Median	9.3
PLT (G/L)	
Range	2-310
Median	65
Lactate dehydrogenase (LDH) U/l	
Range	108-4565
Median	340
Blasts in bone marrow	
<50 %	35
≥50 %	60
Cytogenetics	
Farorable	5
Intermediate	39
Unfavorable	51
Chemotherapy	
Intensive	56
Low dose	27
Best supportive care	12
Molecular tests	Total 60 patients
AML/ETO (positive/negative)	4/56
CBFb-MYH11 (positive/negative)	2/58
NPM1 (positive/negative)	7/53
FLT3/ITD (positive/negative)	13/47
Complete remission	
Yes (total)	51
Yes (after 1^st^ line therapy)	36
No	44
Duration of remission (months)	
Range	2-54
Median	20
Time to relapse (months)	
Range	3-23
Median	12
Survival (months)	
Range	0-55
Median	3

#### Treatment schedules

Fifty six patients were treated with standard induction intensive chemotherapy (daunorubicin plus cytarabine 3 + 7), 27 received low dose chemotherapy (low dose cytarabine or azacitidine) and 12 best supportive care only. After completion of induction therapy response to treatment was evaluated. CR was defined by Cheson criteria. [10]. 40 bone marrow samples were re-evaluated for miR-204 expression after chemotherapy. Patients were followed up for median 21 month (range 1–40 months).

Research was carried out in compliance with the Helsinki Declaration. For the study approval of Bioethical Committee of Wroclaw Medical University was obtained. Written informed consent for study was obtained from all the participants.

### Isolation and expression analysis of microRNAs

Bone marrow mononuclear cells (PBMC) were isolated by Ficoll-Hypaque density gradient centrifugation. Total RNA and microRNA were extracted from collected AML mononuclear cells using mirVana™ miRNA Isolation Kit (Ambion) according to the protocol of the manufacturer. Then 5 μl total miRNA was used as a template into synthesis of cDNA using TaqMan MicroRNA Trasncription Reaction Kit (Applied Biosystems) and 3 μl specific miRNA primers from the TaqMan MicroRNA Assays (Applied Biosystems). Individual reaction was carried out in 15 μl total volume in thermal condition: 16 °C for 30 min, 42° for 30 min, 85 °C for 5 min. TaqMan MicroRNA Assays for miR-204 (hsa-miR-204), and RNU48 were used. The expression level of each microRNA was measured in relative real-time PCR method using TaqMan Gene Expression Assays and TaqMan Fast Universal PCR Master Mix (Applied Biosytems). All reactions were done in triplicate in a total volume of 20 μl on 96-well plates. The real-time PCR was performed on 7900HT Fast Real-Time PCR System (Applied Biosystems) under thermal cycling conditions: 20 s at 95 °C and 40 cycles of 1 s at 95 °C and 20 s at 60 °C. For quantification, the samples were normalized against the expression of RNU48 (internal control). Relative quantification factors (RQ) for the examined miRs were calculated using ^ΔΔ^CT method.

### Statistical analysis

The differences in means of gene expressions between the study and the control patients were estimated using t-Student’s test (for independent samples). To examine the time it takes for death and remission to occur, a Cox’s regression was applied [11]. The difference between the gene expressions before and after treatment was estimated using robust regression and multivariate approach [12]. The computation was performed in R software [13] and based on the simulation technique known as Gibbs sampling in WinBUGS platform [14]. Kaplan-Meier survival curves were used to determine any significant relationship between miR-204 expression and clinical outcome. Results were considered statistically significant when p was <0.05.

## Results

We compared AML samples to healthy controls and found significantly lower miR-204 expression in AML patients (*p* < 0.05). There were no differences in miR-204 expression between male and female patients.

**Fig. 1:**
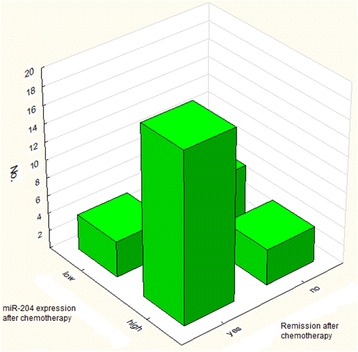
Correlation between miR-204 expression after chemotherapy and remission

After successful induction chemotherapy expression of miR-204 significantly increased (median 0.443606; *p* = 0.000590). Patients with increased miR-204 expression after induction therapy had higher chance for remission (*p* = 0.01438). 83 % of pts with CR after induction therapy had high miR-204 expression and 60 % of patients who did not respond to therapy had low miR-204 expression (Fig. 1). At the moment of diagnosis, Mean miR-204 expression in group who achieved CR was significantly lower than in the group which did not achieve response.

**Fig. 2:**
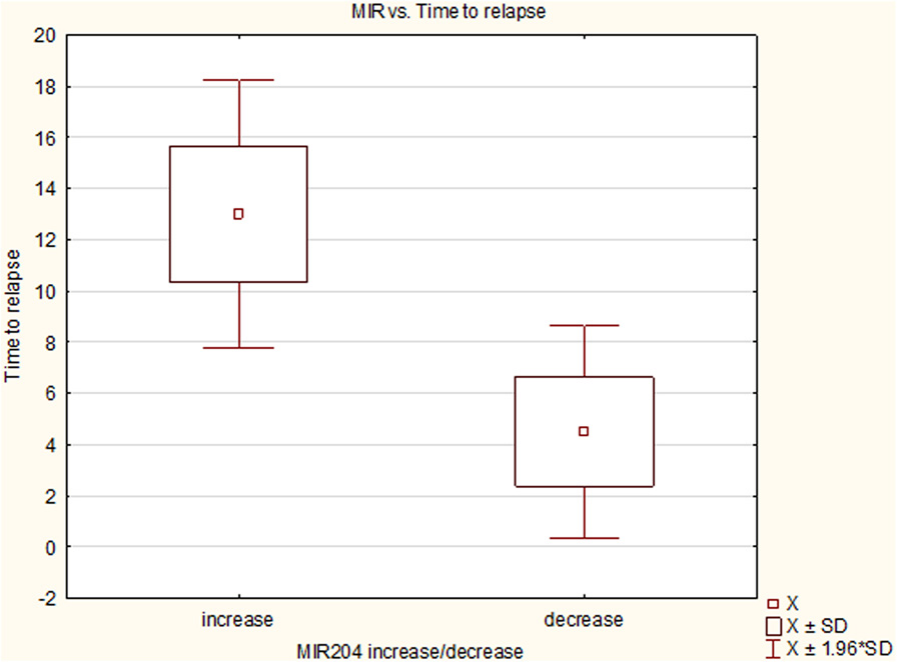
miR-204 expression after chemotherapy and time to relapse

In patients with increase of miR-204 expression after chemotherapy time to relapse was longer (median 13 months) than in patients with decreased miR-204 expression (median 4.5 months), *p* = 0.003347, Fig. 2.

**Fig. 3:**
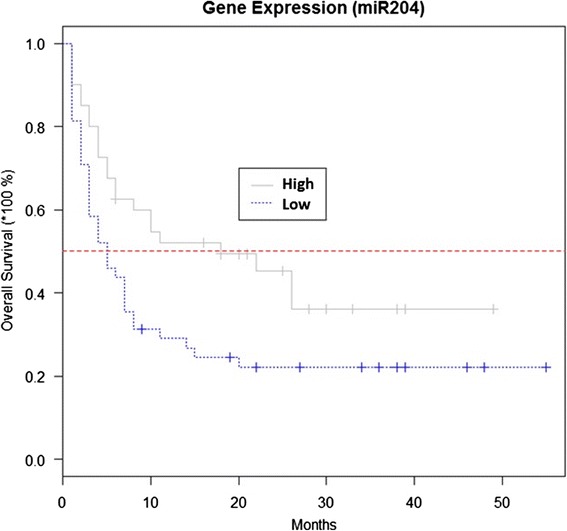
Predicted overall survival depending on miR-204 expression before chemotherapy

miR-204 expression level also influenced patients’ outcome (median value was used as a cut-off). Higher miR-204 expression before therapy was associated with longer survival (Fig. 3).

## Discussion

Small non-coding microRNAs affect process of formation, development, proliferation and apoptosis of normal and malignant cells in human body. Their role has been proved in many cancers, including leukemia [4, 15–17]. Pathways regulation based on microRNA expression is still unknown. Some miRs may act as tumor suppressors and others as oncogenes [18, 19]. Recently, many miRs have been investigated as prognostic and predictive markers. miR-204 expression and its role has been studied in some solid tumors: pancreatic, gastric, prostate [3, 9, 20], in which it acts by down-regulation of Bcl-2 and has tumor suppressor role. miR-204 also influences NTRK2 gene expression in neuroblastoma cancer [21] and FOXC1 gene in invasive endometrial cancer [22].

In our study we showed down-regulated expression of miR-204 in acute myeloid leukemia patients comparing to healthy controls which is in line with observation made by Garzon et al. [23]. Authors found lower miR-204 expression in nucleophosmin positive AML patients and assumed hypothesis, that miR-204 targeted HOXA10 and MEIS1. Those two genes perturb myeloid differentiation and can lead to AML. Ying et al. in their study revealed that loss of miR-204 promotes cancer cell migration through increased expression of brain derived neurotrophic factor or its TrkB receptor [24]. In gastric cancer loss of miR-204 expression was associated with poor outcome, because it caused increase of antiapoptotic protein Bcl-2. In the light of these results Chen et al. demonstrated that miR-204 was down-regulated and its overexpression leaded to loss of cancer cell viability in pancreatic cancer [3]. Authors also showed that miR-204 regulates expression of Mcl-1 (Myeloid cell leukemia) by direct binding to 3′UTR [3]. Overexpressed Mcl-1 is associated with cell survival, while its downregulation leads to cell death. Increased miR-204 negatively regulated Mcl-1. Those observations could explain results of our study. We also analyzed correlations between miR-204 at diagnosis and after chemotherapy and clinical outcome. After effective induction chemotherapy we observed increased miR-204 expression and this change correlated positively with chance for remission achieving. Supposedly higher miR-204 expression could induce leukemic cell deaths and leaded to disease remission. On the other side, patients who had higher miR-204 expression at diagnosis had more favorable clinical outcome than others. Similar role of miR-204 has been also proved in gliomas, where low miR-204 expression leaded to a stem cell-like phenotype, and its overexpression resulted in reduced tumorigenicity and loss of stemness transcription factor SOX4 [24]. In contrast were results by Sümbül et al., who detected high miR-204 expression in colorectal cancer in comparison to healthy population. This finding was not related to any clinicopathological parameters nor survival [9]. Authors explained discrepant findings by the role of increased miR-204 in autophagy and apoptosis.

Concluding, as to our knowledge, we found for the first time down-regulated miR-204 expression in acute myeloid leukemia patients with its implication to disease prognosis. Functionality of miR-204 acting as a tumor suppressor makes this new molecule an useful biomarker in cancer diagnosis and management. Further investigation on miR-204 regulation and its target genes should be performed.
